# Systematic Identification of Anti-Fungal Drug Targets by a Metabolic Network Approach

**DOI:** 10.3389/fmolb.2016.00022

**Published:** 2016-06-17

**Authors:** Martin Kaltdorf, Mugdha Srivastava, Shishir K. Gupta, Chunguang Liang, Jasmin Binder, Anna-Maria Dietl, Zohar Meir, Hubertus Haas, Nir Osherov, Sven Krappmann, Thomas Dandekar

**Affiliations:** ^1^Department of Bioinformatics, Biocenter, University of WürzburgWürzburg, Germany; ^2^Microbiology Institute – Clinical Microbiology, Immunology and Hygiene, Friedrich-Alexander University Erlangen-Nürnberg, University Hospital of ErlangenErlangen, Germany; ^3^Division of Molecular Biology/Biocenter, Medical University InnsbruckInnsbruck, Austria; ^4^Aspergillus and Antifungal Research Laboratory, Department of Clinical Microbiology and Immunology, Sackler School of Medicine, Tel Aviv UniversityTel-Aviv, Israel

**Keywords:** metabolism, targets, antimycotics, modeling, structure, interaction, fungicide

## Abstract

New antimycotic drugs are challenging to find, as potential target proteins may have close human orthologs. We here focus on identifying metabolic targets that are critical for fungal growth and have minimal similarity to targets among human proteins. We compare and combine here: (I) direct metabolic network modeling using elementary mode analysis and flux estimates approximations using expression data, (II) targeting metabolic genes by transcriptome analysis of condition-specific highly expressed enzymes, and (III) analysis of enzyme structure, enzyme interconnectedness (“hubs”), and identification of pathogen-specific enzymes using orthology relations. We have identified 64 targets including metabolic enzymes involved in vitamin synthesis, lipid, and amino acid biosynthesis including 18 targets validated from the literature, two validated and five currently examined in own genetic experiments, and 38 further promising novel target proteins which are non-orthologous to human proteins, involved in metabolism and are highly ranked drug targets from these pipelines.

## Introduction

The treatment of invasive fungal infections caused by the versatile saprophytic fungus *Aspergillus fumigatus* is challenging (Denning, 1998). While the healthy human immune system is able to fend off *A. fumigatus* infections in general, immune-deficient patients are highly vulnerable against invasive aspergillosis. Aspergillosis is one of the major lethal conditions in immunocompromised patients (Dagenais and Keller, 2009). In eukaryotic pathogens, most potential protein targets for antimycotic development bear a considerable risk of toxic side effects for the patient as a similar protein might be present in the human host.

Although several anti-mycotic strategies exist, they are only partially effective due to the significant immunosuppression of those patients. Therefore, the development of new therapeutic strategies against *A. fumigatus* infection is crucial.

Targeting the metabolism of pathogens is in general a valid strategy as it is central for pathogen survival and there is also a lower chance for development of resistance mutations as those usually affect fitness and are thus counter selected (Kohanski et al., 2010).

Unlike many other approaches that exploit a direct anti-fungal therapy pursuing identified antimycotic leads, we want to introduce here a novel, general strategy to tackle a pathogen at the metabolic level, choosing the human-pathogenic mold *A. fumigatus* as example. Known challenges in the search for new antimycotic targets include the high similarity between fungal genes and those of the human host. To minimize this problem, we combine three different bioinformatics approaches that we have previously developed to target the pathogen's primary metabolism: (I) metabolic modeling (for instance applied to *S. aureus* antibiotics in Cecil et al., 2015): direct metabolic network modeling using elementary mode analysis and flux estimates constrained by applying gene expression data, (II) enzyme regulation-based strategy: targeting metabolic genes by transcriptome analysis of condition-specific highly expressed enzymes (for instance applied to *S. aureus* antibiotics in Cecil et al., 2011), (III) protein-protein interaction-based strategy: analysis of enzyme structure, enzyme interconnectedness (“hubs”) and identification of pathogen-specific enzymes using orthology relations (for instance applied in viral infections in Shityakov et al., 2015).

Each of these approaches has its strengths and limitations, however, their combination offers a powerful tool to reveal metabolic targets for later drug development. Based on the resulting candidates we suggest a prioritized list of target genes that are important for *A. fumigatus* but have no close orthologs in humans. By focusing the effort on the metabolic pathways for (a) vitamin synthesis, (b) lipid biosynthesis, and (c) amino acids biosynthesis, we developed a pipeline that integrates and compares results from all three bioinformatics approaches (I-III) to reduce and focus the target list to the most promising candidate genes.

These candidate proteins for targeting fungal metabolism by antimycotics were in part validated according to literature evidence, several are currently tested and evaluated experimentally while others are still available for targeting. Additional information can be incorporated for further refinement and iterations of our combined target screening pipeline. Our workflow is not restricted to *A. fumigatus* but can also be easily transferred to other pathogens which are similar challenging to target.

## Materials and methods

### Metabolic modeling

**Figure 1:**
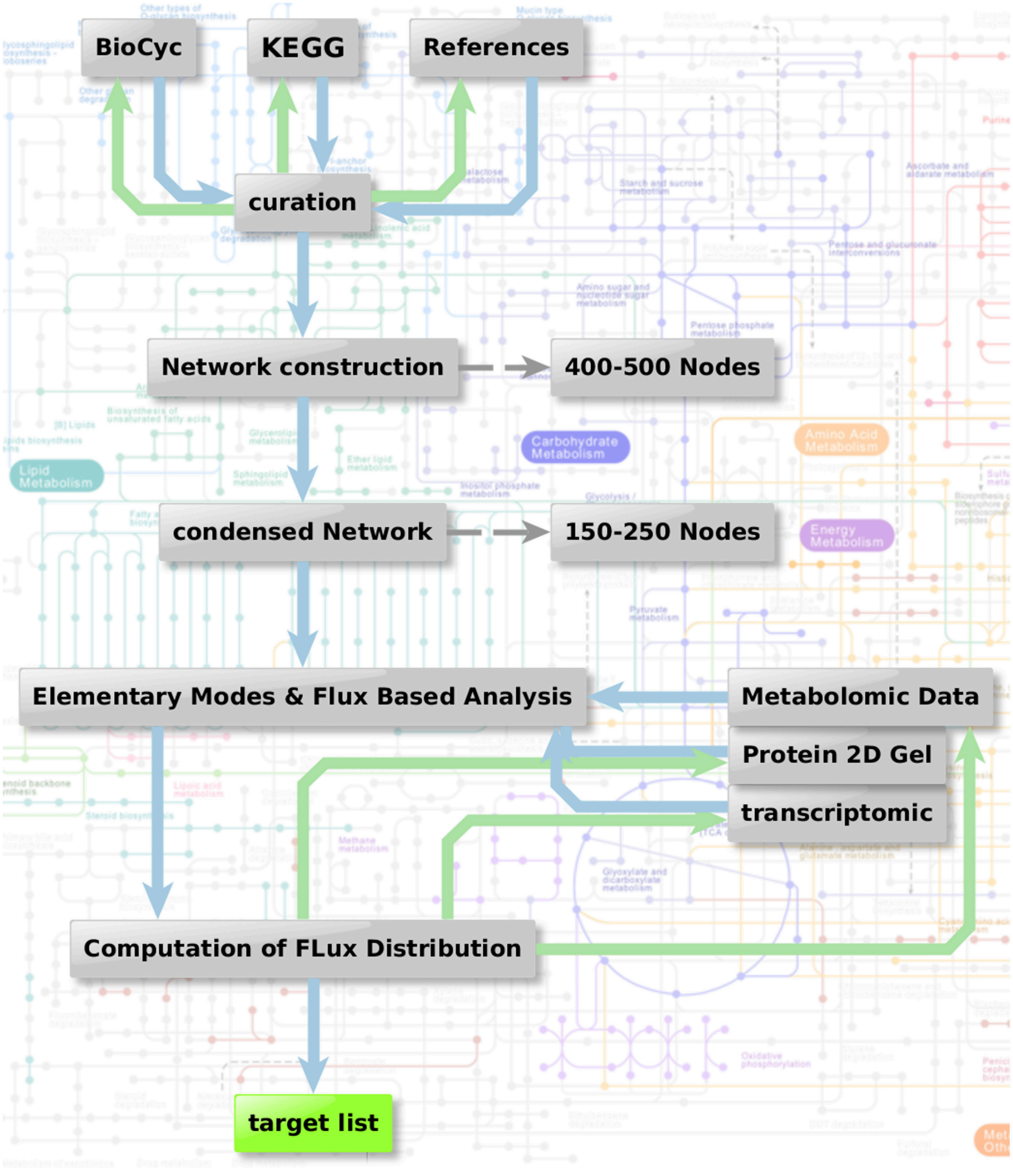
**Metabolic network modeling strategy**. Flow chart of analysis procedure to obtain a metabolic network which can be used for the prediction of flux and elementary modes in context with the help of various different types of data. Two steps are combined: Elementary mode analysis gives an overview on all metabolic pathways accessible for the pathogen. Based on this network, algorithms such as YANA, YANAsquare, and YANAvergence offer different routines to calculate implied flux value for different pathways using gene expression data, protein expression data or metabolite concentration changes. Targets which are critical in a metabolic sense are easily identified considering hub enzymes, enzymes with a high metabolic control and observation of flux change in general.

Figure 1 shows the metabolic network modeling approach applied as a first strategy to target fungal metabolism by interfering substances. We outline the flow chart of analysis procedures used to obtain a metabolic network which can be used for prediction of flux and elementary modes with the help of various different types of data and software.

As a first step we apply a process of pathway reconstruction that identifies the *A. fumigatus* enzymes involved in the different pathways.: For this we used biochemical databanks such as KEGG (Kanehisa and Goto, 2000; Kanehisa et al., 2014), Roche pathways (Michal and Schomburg, 2012), and Metacyc (Caspi et al., 2016). Sometimes data from this source are not complete or incorrectly annotated. Such knowledge gaps were filled by literature and expert knowledge including sequence analysis and reannotation of incorrect annotations. The complete primary metabolism from *A. fumigatus* was modeled in this way to yield a metabolic network, including the major carbohydrate metabolism (glycolysis, pentose phosphate pathway, TCA cycle), nucleotide biosynthesis, amino acid biosynthesis and degradation, and fermentation pathways.

Furthermore, cofactors and cell wall synthesis were also taken into account. Moreover, we created sub-networks for vitamins and fatty acid metabolism. For these additional components we looked only at selective reactions and pathways, which we considered particularly promising for targeting and being absent in humans. Accordingly, we selected basic fatty acid metabolism, ergosterol, and glucan metabolism, as well as vitamin metabolism with focus on riboflavin and thiamine.

#### Elementary mode analysis

A method to identify metabolic pathways that might be crucial for growth is the elementary mode analysis. A flux mode is a set of enzymes, which balance all metabolites within a metabolic network such that these “internal metabolites” do not accumulate or are diminished (not considering sources and drains, the “external metabolites”). Those flux modes (combinations of enzymes) which cannot be decomposed further without affecting this balance are called elementary modes. We computed these elementary modes using the Metatool program (von Kamp and Schuster, 2006), which has been integrated within the YANA software package (Schwarz et al., 2005). Further analysis of the calculated pathways (i.e., all elementary modes) considered which metabolic enzymes are valid antibiotic targets. These are enzymes that are essential for the metabolism as without their operation there are no alternative routes available to produce critical metabolites required for growth.

#### Flux mode strength

To calculate condition-specific strengths of different metabolic fluxes, large-scale transcriptome data-sets were used as constraints to fit the metabolic model with the aim to estimate flux distributions (Schwarz et al., 2005, 2007) for optimal growth conditions and changes under biofilm condition. The training procedure and algorithms involved (Gradient descent: BFGS–Boyden-Fletcher-Goldfarb-Shannon optimization method) have been described previously (Liang et al., 2011).

In summary, we use two methods sequentially: First, elementary mode analysis provides an overview of all metabolic pathways accessible for the pathogen. Second, based on this network, algorithms such as YANA, YANAsquare, and YANAvergence offer various routines to calculate implied flux values for different pathways using gene or protein expression data. Changes in metabolite concentration are best to calculate by flux analysis, but being elaborate to measure and hence seldom available, they were therefore not available here. Both methods identify those metabolic enzymes which are essential for growth, either as they are involved in unique routes to provide metabolites required for growth (elementary mode analysis) or as they carry a strong metabolic flux, either constantly (housekeeping enzymes) or in the relevant situation of invasion and infection.

### Enzyme regulation-based strategy

**Figure 2:**
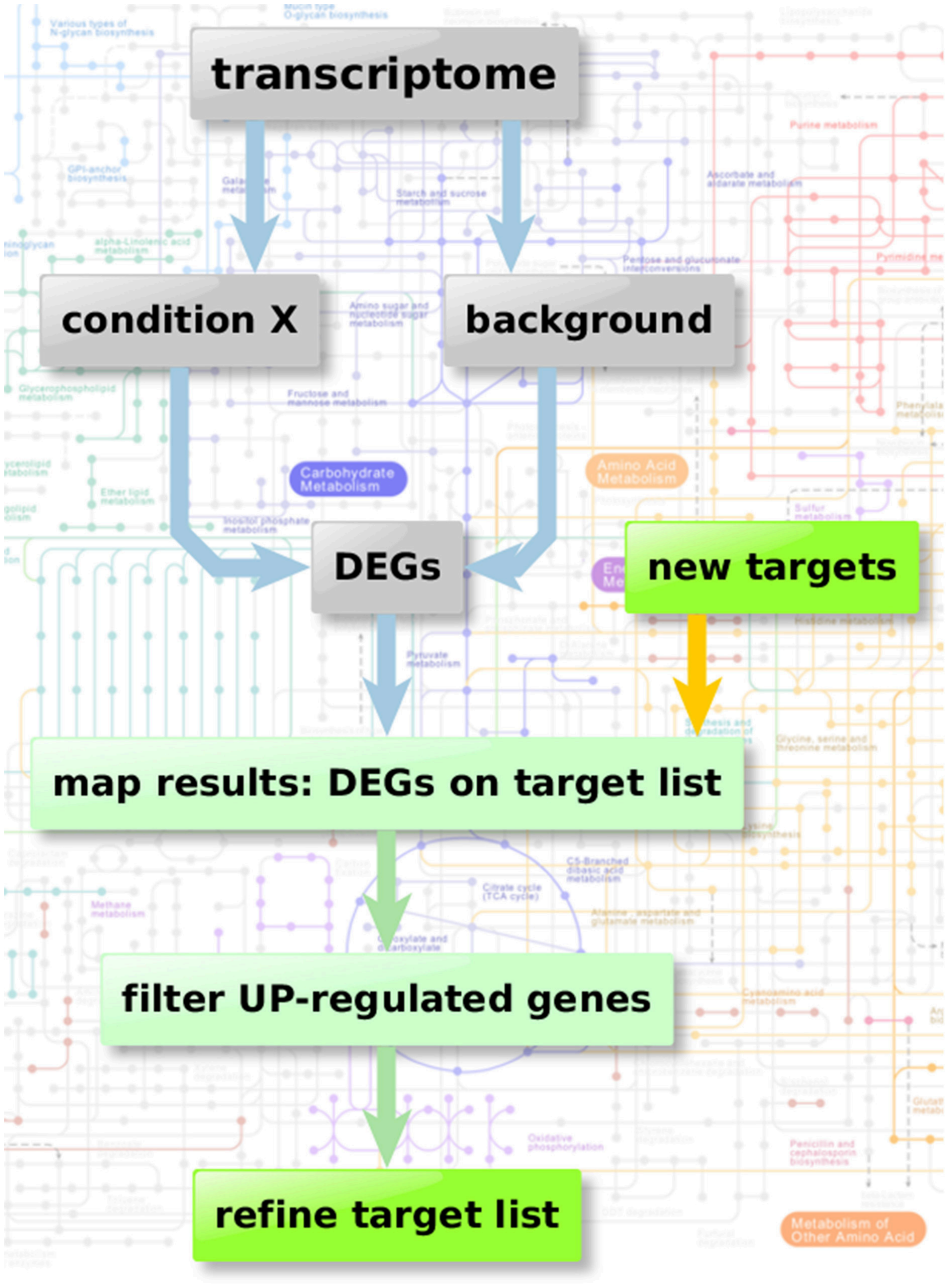
**Enzyme regulation-based strategy**. Flow chart of transcriptome analysis to verify the quality and potential of the suggested genes for new anti-fungal therapeutic strategies. Starting with transcriptome data two different sample groups were defined: condition X marking the specific experimental significant genotype/sample type and a group of background samples. The comparison results in a list of differential expressed genes in “condition X” which are considered to be important in the specific condition. Those differential expressed genes can then be mapped on the list of potential targets resulting from the scoring and filtering procedure. Those genes aligning with the target list is filtered regarding up-regulated genes (URGs) which are considered as promising targets for knockout and thereby as targets for new therapeutic strategies.

Figure 2 depicts the flow chart of the enzyme regulation-based strategy. Transcriptome datasets we used for our comparison analysis involved: Bruns et al. (2010) (accession GSE19430); Schrettl et al. (2010) (accession GSE22052); Willger et al. (2008) (accession GSE12376). These data sets were available from Gene Expression Omnibus (Edgar et al., 2002; Barrett et al., 2013), a database collection of extensive experimental gene expression results as well as from additional experimental sources (McDonagh et al., 2008). We analyzed the datasets using the GEO2R framework, a built-in function of the GEO database, which uses the R statistics software (Davis and Meltzer, 2007; Huber et al., 2015; R Core Team, 2015; Ritchie et al., 2015) for normalization, fitting, comparison, and visualization of provided microarray data and calculates the resulting differential expressed genes.

After identification of relevant experiments to reveal key enzymes involved in metabolic adaptation as prospective antimycotic targets we defined two different groups of samples (background and relevant condition).

Relevant experiments concerned here available GEO datasets on *A. fumigatus* genes involved in virulence. Such genes have to be induced by appropriate conditions, we found the following datasets: changes during invasion initiation (McDonagh et al., 2008), by iron deficiency (Schrettl et al., 2010) and under hypoxia adaptation (Willger et al., 2008).

We next compared the expression level of all genes. Using GEO2R we calculated logarithmic fold change (logFC)-values as well as *p*-values for every gene. We modified the GEO2R output in the manner that the output only consisted of genes with a *p* < 0.05. Those genes were considered as significantly differential expressed genes under given conditions.

In the next step, the resulting list of differential expressed genes was matched onto the list of potential targets (all *A. fumigatus* metabolic enzymes; later enriched subsets) to receive the relative expression of those target genes in comparison to control conditions.

The control condition was here in all comparisons the wild type of *A. fumigatus* grown in rich medium with wild type strains used as control being: AF293 for the data set analyzed from Schrettl et al. (2010) and McDonagh et al. (2008); CEA10 in Willger et al. (2008)).

Furthermore, we analyzed the resulting expression list of target genes with respect to increased transcript level since up-regulated genes (URGs) are considered important genes for *A. fumigatus* growth under this condition, thus indicating potential antimycotic targets. Additionally we compared those up-regulated target genes with the metabolic simulation data to specify the quality of each suggested target.

### Protein-protein interaction-based strategy

**Figure 3:**
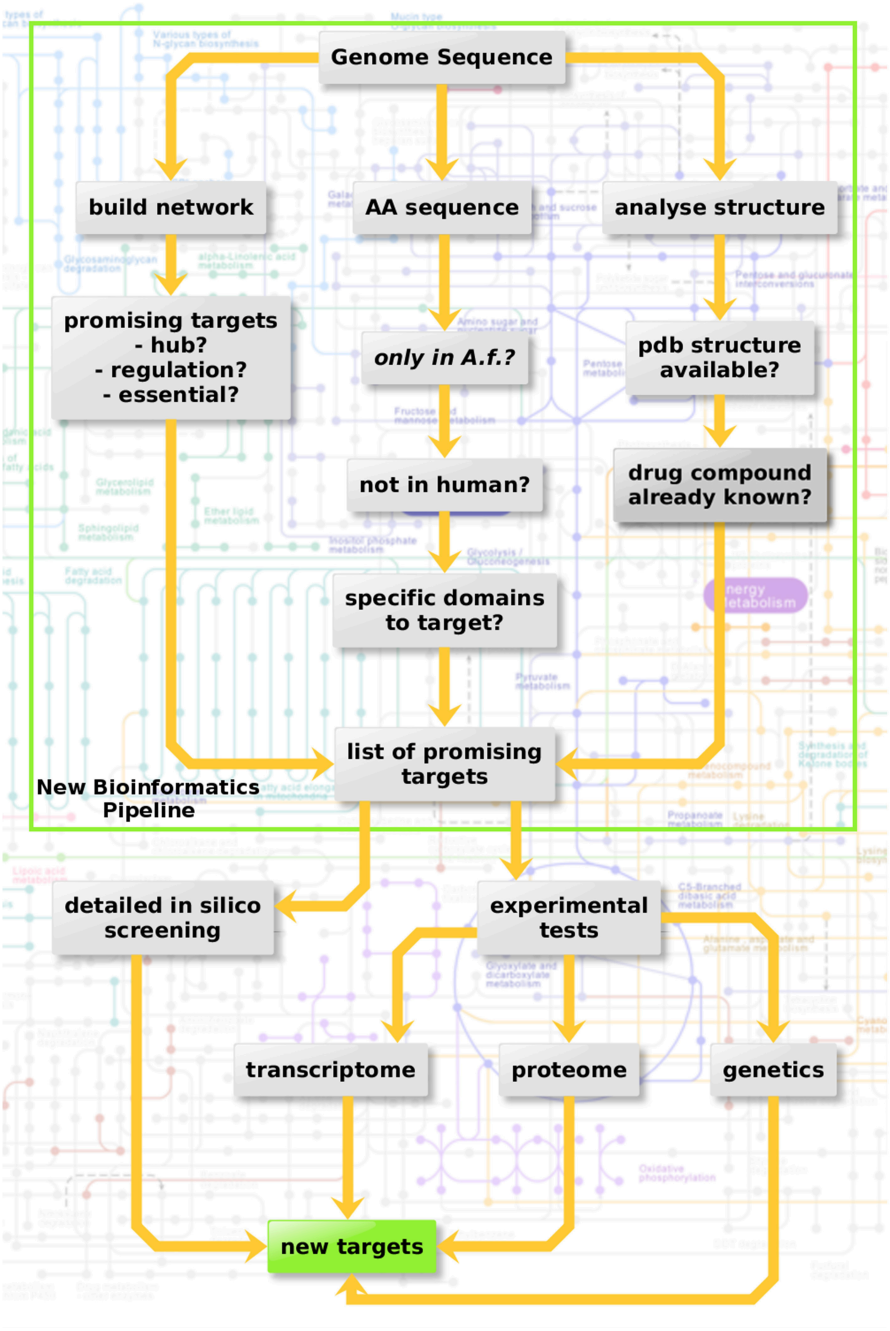
**Protein-protein interaction-based targeting of metabolism**. Three criteria are combined here for in silico screening (top): Metabolic network hub or rim protein, targetable metabolic or regulatory domains which have no orthologue in the host as well as available pdb structure and drug targeting the structure.

Three criteria were considered here in three sub-pipelines for *in silico* screening (Figure 3, top): Metabolic network hub or rim protein, targetable metabolic, or regulatory domains which have no ortholog in the host as well as available structure from the protein databank and drugs targeting the structure.

#### Sequence comparisons

Sequence comparisons used standard techniques such as basic local alignment sequence tool (Altschul et al., 1997). We used the following parameters: A stringent *e*-value threshold 1e-10, a bit-score of over >500, word_size 6, scoring matrix Blosum62, gap existence cost 11, gap extension cost 1 for the blastp based similarity searches followed by manual inspection of functional domains and query coverage to avoid misinterpretation.

#### Protein interaction data

Protein interaction data were taken from the DIP database (Database of Interacting Proteins; Salwinski et al., 2004). The orthologous protein sequences from *A. fumigatus* were mapped on the available interaction data from DIP using the interolog approach. All the orthologous interactions were predicted with OrthoMCL (Li et al., 2003; Fischer et al., 2011). The resulting interactions in *A. fumigatus* were further investigated to check which of them are supported by domain-domain interactions (DDI) using the DDI containing databases DOMINE (Yellaboina et al., 2011), DIMA 3.0 (Luo et al., 2011), and IDDI (Kim et al., 2012). The subcellular localization was predicted using an improved KnowPred software (Lin et al., 2009), the latest UniLoc server and SwissProt localization annotations for these proteins (bioapp.iis.sinica.edu.tw/UniLoc/). The localization information was further used as confirmatory evidence for plausible interacting proteins. Only the domain that supported interactions in which the interacting partners shared a minimum of one similar localization was considered as true protein-protein interaction (PPI) in our reconstructed *A. fumigatus* interactome. The protein interaction network was reconstructed using Cytoscape (Smoot et al., 2011). Network analysis predicted highly connected nodes (hubs) and metabolic bottlenecks which suggested topologically important proteins which subsequently can be used as potential drug target if they do not have homology with human proteins. Of the top 20% proteins based on degree rank and betweenness rank were retrieved and re-ranked based on the minimum of their cumulative rank in the common top 20% proteins S

$$\begin{array}{l} {\text{X}}_{\text{r}}=\sum {\text{X}}_{\text{de}}+{\text{X}}_{\text{be}} \end{array}$$

Where X_r_ is the new rank of protein X, the index “de” refers to the degree rank of protein X and the index “be” refers to the betweenness rank of protein X.

Metabolic bottlenecks are pathways and reactions for which no alternative routes exist, so all metabolic flux for the following metabolites has to go through such a bottleneck; these points are also called “choke points” (Rahman and Schomburg, 2006) and are a valid and promising position to interfere with primary metabolism.

Betweenness centrality looks how central a given protein (“vertex” or “node”) is in a network. It counts the number of shortest paths from all vertices to all others that pass through that node. The degree rank just counts how well connected a protein target is by counting all edges leading to one of the vertex (so how many interactions converge on this protein). Further details are found in Thadakamalla et al. (2005).

The newly ranked protein were further compared in their sequence against the human proteome and the proteins showing significant similarity with human were discarded from the potential drug target list. The KEGG (Kanehisa and Goto, 2000; Kanehisa et al., 2014) database was used to annotate the proteins in the target list that have involvement in fungal metabolism.

### Drug targets with orthology to functionally important genes

Furthermore, the *A. fumigatus* proteome and eukaryotic proteins available at Database of Essential Genes (DEG) (Luo et al., 2013) were analyzed to identify orthologous protein using OrthoMCL (Li et al., 2003; Fischer et al., 2011). We further applied a BlastP (Altschul et al., 1997) based screening to filter out *A. fumigatus* protein in the set of orthology-based predicted important proteins that are significantly similar with human proteins. Next, the interacting as well as predicted essential proteins were metabolically annotated to establish the metabolic importance and metabolic pathway involvement. The final list of potential drug target were scored and ranked accordingly to the RhumPdb score (Toomey et al., 2009).

$$\begin{array}{l} R_{humDB}={\text{log}}_{10}\left(\frac{E_{BlastP}\;\left[query\;vs\;human\;proteome\right]}{E_{Blastp}\;\left[query\;vs\;PDB\right]}\right) \end{array}$$

A high *R*_*humPDB*_ score indicates that the target has minimum similarity with human and has a close protein data bank (*PDB*) structure template.

To identify and prioritize targets in the metabolism of *A. fumigatus*, first all metabolic reactions were mapped using the orthology information from the already available metabolic models of *Aspergillus oryzae, Aspergillus niger*, and *Aspergillus nidulans* (models by Andersen et al., 2008; David et al., 2008; Vongsangnak et al., 2008). Furthermore, reactions that were not annotated from the orthology were fetched using the Blast2GO (Conesa and Götz, 2008) annotation and Enzyme database (Bairoch, 2000). At every step manual verification was performed to remove any redundant information. The preliminary list of metabolic reactions was reduced based on the following criteria: (i) all those reactions which were catalyzed by true orthologs of human proteins were removed; (ii) the list was compared to the DEG database (Luo et al., 2013) of orthology based genes to ensure that no gene potentially vital for growth was removed from the list during the reduction process; (iii) for the enzymes also present in the interactome, the number of protein interactions these proteins have (“degree”) was also considered during reduction as an enzyme with high degree (interacting with many proteins, connected to many pathways) has more possibility to be involved in multiple pathways and *vice versa*; (iv) as a key simplification, and to avoid combinatorial explosion during elementary mode calculation, we selected very few enzymes for any linear stretch while considering the metabolites which are similar; (v) the pace-maker (flux value determining) enzymes for long linear pathways were generally included; (vi) finally, pathway annotation was done for this list and only the reactions which participate in primary metabolic pathway were further considered to find the drug targets in primary metabolism. Moreover, we calculated the RhumPDB score (Toomey et al., 2009) and mapped the gene expression data (Bertuzzi et al., 2014) over the preliminary target list to prioritize the drug targets.

The following criteria were used to prioritize targets further: (i) genes that were highly expressed at many independent time points were given top priority (mean significant differential expression) over the genes highly expressed at fewer time points. A top expression rank represents a higher expression of a gene at all time points during invasive infection (ii) the final priority order was decided based on the minimum of RhumPDB (Toomey et al., 2009) and expression rank (iii) it was also noted whether the enzyme participates in a fungal-unique pathway or any pathway is shared with human metabolism. Proteins were ignored for which co-ortholog proteins were available, as this implies that the same reaction might be catalyzed by alternative proteins.

## Results and discussion

### Targets from metabolic modeling to interfere with pathogen proliferation

We defined here that the targets which are critical in a metabolic sense to be easily identified by pointing out strong active pathways pertaining to different environmental conditions. Calculating the resulting differential effects on metabolite synthesis and growth then allows the identification of enzymes which are essential for growth and valid drug targets. To achieve a compact network regarding identification of new antimycotic targets in *A. fumigatus*, first we removed many non-central *A. fumigatus* metabolic reactions in vitamins, lipids, and amino acid metabolism that are governed by human orthologous proteins. The central carbohydrate metabolism consisting of glycolysis, PPP and TCA, however, was retained, as otherwise no flux calculations covering a major part of central metabolism are possible. We then used only those reactions for which we had availability of gene expression data during infection. Overall, the enzymes we selected in the analysis were based on their importance in metabolic pathways, pathway crosstalk and flux activity analysis in the model. With these criteria for filtering the resulting list consists of 162 unique metabolic reactions which were specific for *A. fumigatus* and that are catalyzed by 102 enzymes and contained 204 metabolites.

**Figure 4:**
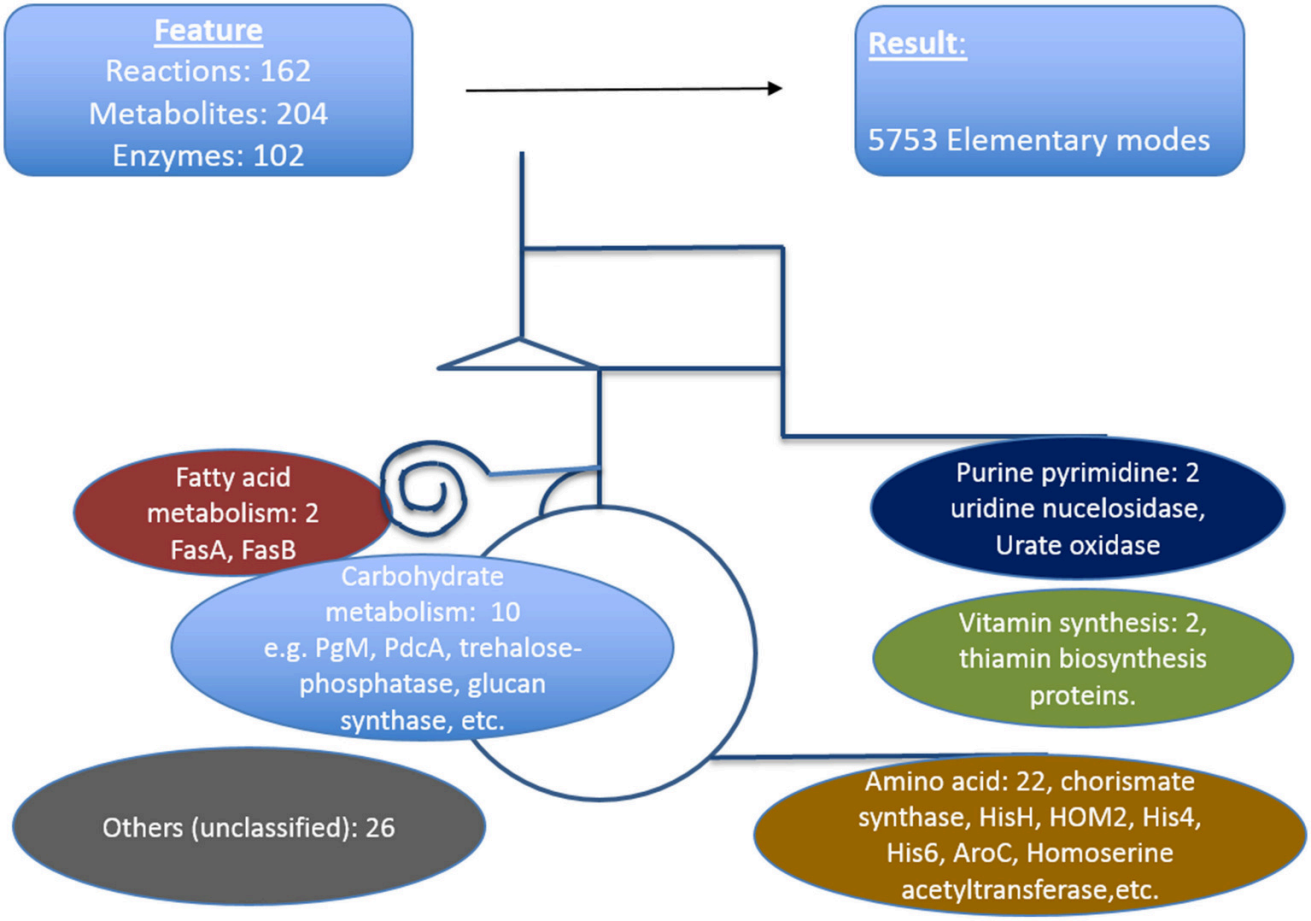
**Metabolic model of ***A. fumigatus*****. This figure illustrates the metabolic web considered, focusing on primary metabolism. Top: Shown are reactions, metabolites and enzymes (left) and modes calculated (right). Below major pathways modeled are given together with the number and examples of identified promising antibiotic target structures. Details are found in Supplementary Material File 19 together with input files, stoichiometric matrix, calculated elementary modes, and flux values for optimal growth and biofilm condition.

The resulting model of central primary metabolism for *A. fumigatus* is illustrated in Figure 4, and given in full detail in the Supplementary Material (input files for calculations with Metatool see Supplementary Table 1; all enzyme reactions considered are also given (Supplementary Table 15, with the mapping to gene identifiers given in Supplementary Table 19) so that from the stoichiometric matrix [Supplementary Table 16 or Supplementary Table 18 (null space version)] all elementary modes (Supplementary Table 17) can be calculated).

The model contains the central carbohydrate and amino acid metabolism, lipid synthesis and degradation as well as key reactions for intermediary metabolism and all necessary cofactors, often vitamins. From a systems biology point of view there are hub metabolites (such as currency metabolites including ATP, but also pyruvate, glutamic acid), central enzyme nodes (for instance adenylate kinase to balance energy), unique pathways and reactions, which are similar as “choke points” (Rahman and Schomburg, 2006) to represent good positions for interfering with metabolism. Overall, our network involves 204 metabolites, 102 enzymes, and 5737 elementary modes (model in YANA/SBML format: Supplementary Table 20). Such a model serves as a basis for both targeting genes encoding metabolic enzymes as well as phenotyping with respect to growth or nutritional characteristics. Phenotyping including prediction of essential genes for growth requires a large model where most metabolic reactions in these pathways and their branching are considered so that there is good correlation between prediction and any observed phenotype. Furthermore, larger models allow identification of well-connected enzymes socalled hub enzymes. Further methods such as metabolic control theory allows to identify enzymes with a high metabolic control coefficient as well as refined modeling of flux changes in general.

Specific fluxes and changes, were calculated using YANA: As starting values for the flux calculation the flux value for each elementary mode was set to 1; gene expression data sets were next used to calculate affected key modes: The different expression values for each enzyme were used to coarsely approximate their different activity levels. Systematically different flux values for each elementary mode were combined using a genetic algorithm (Schwarz et al., 2005) and steepest descent methods (Cecil et al., 2011) to minimize the error of the squared differences between calculated and approximated enzyme activity. Integration of gene expression data provides an acceptable way to calculate fluxes for those parts of the metabolic network which cannot be validated by directly measured metabolic data. We know that the average error for the flux value calculated from gene expression data is around 5–10% for the network, if a large scale data set is available and used on the pathways of interest and the simulation converges. This was validated by looking at and measuring differences in metabolite concentrations for the calculated flux values in several of our studies in infection biology (Cecil et al., 2011, 2015).

Recent studies have shown that the fungus *A. fumigatus* in its aerial grown biofilm-like state exhibits reduced susceptibility to antifungal drugs and undergoes major metabolic changes that are thought to be associated with virulence. These differences in pathological and physiological characteristics between biofilm and liquid shake conditions strengthen the notion that the planktonic state condition is a poor *in vitro* disease model. We therefore also used actual biofilm gene expression data from Muszkieta et al. (2013) for comparative calculations to the planktonic state datasets. Calculations were performed with YANAvergence as detailed in Materials and Methods to provide the resulting planktonic state fluxes (Supplementary File 2) and the gene expression under biofilm conditions (Supplementary File 4). That convergence was achieved and the data of the predicted fluxes correlate well with the gene expression data is shown in Supplementary File 3 (for the planktonic state) and Supplementary File 5 (for biofilm formation). Note that this calculation also reveals which enzymes in *A. fumigatus* are growth condition-specific higher or lower active (regarding their fluxes) as predicted just according to the gene expression data (all the points which are above the diagonal show higher gene expression (“experiment”) then their calculated flux; see Supplementary Files 3, 5). Points below the diagonal indicate stronger flux then expected from the experimental data on gene expression. The detailed datasets are provided in Supplementary File 2 (planktonic growth, left: enzyme name, mapping to gene identifiers is given in Supplementary Table 19; middle: flux values compared to gene expression, right and the resulting regulatory difference, very right) and Supplementary File 4 (biofilm growth).

#### Target ranking and identification

According to the metabolic modeling we performed, pathways differentially regulated correlating with the biofilm gene expression datasets include: glycolysis (down-regulated) and gluconeogenesis (up-regulated, see for example: enolase flux is in the correct direction); down-regulatedlipid, fatty acid, and isoprenoid metabolism up-regulated, in particular ergosterol and cell wall synthesis, pentose phosphate cycle, nucleotide metabolism (signaling molecules), amino acid metabolism up-regulated and vitamin and cofactor metabolism down-regulated, several degradation pathways upregulated (Table 1). Such strongly induced genes qualify (see M&M; prioritization of targets) as potentially interesting targets for antimycotics as they are essential for growth, emerging from this approach as these enzymes carry a strong flux under biofilm formation (in that sense these are named “pace maker enzymes” as they determine the flux value and how strong this pathway is active). The Table 1 only provides examples for the top enzymes identified by this criterion. For optimal results, however, all criteria have to be compared and combined (see Results, part 4). In particular, we also considered from this approach the results from the elementary mode analysis and identified enzymes essential for growth by providing building blocks of primary metabolism required for growth.

**Table 1:** **Top Targets from metabolic modeling to target pathogen metabolism^**a**^**.

**Pathway**		**Target by pace maker enzyme**	**Validated by flux analysis**
Vitamin	Riboflavin	RIB1	Yes
Nucleotide	Pyrimidin	Carbamoyl synthetase	Yes
Lipid	Ergosterol and	Ergosterol synthetase	Yes
	Cell wall synthesis		Yes
Carbohydrates	Glycolysis	Aldolase, enolase	Yes
	Pentose Phosphate Cylce	Transketolase	Yes

a *Summary of pathways and their primary metabolites investigated as well as important enzymes which posses a capability as pace maker in their pathway (middle column) and whether they could be validated by flux analysis, and are therefore considered as prioritized targest. Furthermore, there is evidence from other systems, e.g., overexpression of the first gene of the riboflavin biosynthetic pathway (RIB1) is already sufficient to obtain yellow colonies and the accumulation of riboflavin in the supernatant in Pichia pastoris (Marx et al., 2008). Validation by flux analysis is given in detail in Supplementary Material (Supplementary Tables 2, 4) indicating that the pathway did undergo expression changes comparing the different data sets against each other in the flux calculations*.

#### Targets from an enzyme regulation-based strategy

We next show the flow chart for the enzyme regulation-based strategy (Figure 2). First, we use available transcriptome data to verify the quality and potential of the suggested genes and their resulting proteins as antifungal targets. To analyze transcriptome data two different conditions are usually compared: A specific expression condition X marking the specific experimental significant genotype/sample type and for comparison the control or background samples. The comparison results in a list of differential expressed genes. The user can apply different filters to identify promising targets shared or exclusive for one condition, up- or down regulation and of course the “no orthologs in man” criterion (to avoid side effects in man). Genes which are robustly expressed under many different conditions (true housekeeping genes) represent another possible targeting strategy.

Differential expressed genes can then be placed on the list of potential targets resulting from the scoring and filtering procedure. Those genes aligning with the target list are filtered regarding URGs which are considered as promising targets. The screen for different genes upregulated in the transcriptome for conditions such as iron deficiency (details in Supplementary File 8; Schrettl et al., 2010), hypoxia adaptation (see Supplementary File 7; Willger et al., 2008) and invasion initiation conditions (Supplementary File 6; McDonagh et al., 2008) shows different prominent metabolic genes significantly (*p* < 0.05, multiple testing condition) up- (red) or down-regulated (green) under these condition (Table 2), the control is always wild type. This allows for the identification of genes which are crucial for survival (highly expressed) under those challenging conditions, as an antimycotic will be a similar stress. By targeting the major players involved in metabolic adaptation, we increase the likelihood of a strong inhibitory effect that may stop metabolism, inhibiting all growth and eventually killing the cell. This transcriptome-based target identification has also promise for a quite different strategy, targeting those genes which are critical for maximum growth under optimal conditions (green entries in Table 2).

**Table 2:** **Metabolic gene expression under iron deficiency, invasion initiation, and hypoxia compared to control^**a**^**.

	**Iron deficiency**	***In vivo***	**Hypoxia adaptation**	**Pathway**	**Annotation**
**Sup6**					
AFUA_3G04210	1.052	−1.058	−1.474	FA	Fatty acid synthase alpha subunit FasA
AFUA_3G04220	1.153	−1.971	n.s.	FA	Fatty acid synthase beta subunit
AFUA_3G09290	−0.706	−0.719	1.479	AA	Phosphoglycerate mutase, 2,3-bisphosphoglycerate-independent
AFUA_3G06830	n.s.	−1.684	0.945	AA	Aspartate-semialdehyde dehydrogenase
AFUA_1G14570	n.s.	1.042	−2.718	AA	Phosphoribosyl-AMP cyclohydrolase
AFUA_4G06460	n.s.	2.834	n.s.	AA	Pentafunctional AROM polypeptide
AFUA_3G14490	n.s.	−1.401	−1.574	AA	Ketol-acid reductoisomerase
AFUA_5G05590	n.s.	−1.060	1.703	AA	Aspartokinase
AFUA_5G05820	n.s.	−2.065	1.107	AA	Homoserine kinase
AFUA_6G12400	n.s.	−0.031	−1.568	SUC	1,3-beta-glucan synthase catalytic subunit FksP
**Sup2**					
AFUA_6G11390	−0.926	0.459	−2.196		1,3-beta-glucanosyltransferase Gel2
AFUA_3G11070	0.987	−4.248	n.s.	GLU	Pyruvate decarboxylase PdcA
AFUA_2G10740	n.s.	−1.164	n.s.	VIT	Thiamin biosynthesis protein (Thi-4)
AFUA_5G12190	n.s.	−4.310	−0.817		Transcription initiation factor subunit (TAF30)
AFUA_4G09660	n.s.	1.849	n.s.		Secretory component protein shr3
AFUA_6G12400	n.s.	−0.031	−1.568	SUC	1,3-beta-glucan synthase catalytic subunit FksP
AFUA_1G06700	n.s.	−1.705	n.s.		Metacaspase CasA
AFUA_3G14140	0.367	−1.273	−1.262		Metacaspase CasB
AFUA_4G13340	n.s.	−0.008	−1.412		DUF907 domain protein
AFUA_2G17650	−0.523	2.921	−2.795		DUF907 domain protein
AFUA_2G17300	n.s.	4.050	1.057	GSH	Glutathione S-transferase
AFUA_2G09040	n.s.	1.360	1.053		Vacuolar transporter chaperone (Vtc4)
AFUA_2G04010	n.s.	−1.125	1.182	SUC	Alpha,alpha-trehalose-phosphate synthase subunit
**ESSENTIAL GENES**					
AFUA_3G14440	0.916	−1.174	n.s.		Cytochrome c oxidase family protein
AFUA_4G10480	n.s.	−2.375	1.812		Mitochondrial large ribosomal subunit protein L30
logFC: -1	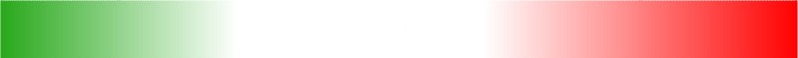			1	

a *Only genes are listed that resulted from two ranking functions (RhumPDB + Expression, Supplementary Table 14; DegreeRank + BetweennessRank, Supplementary Table 10) and additionally show a high logarithmic fold change of >1.0 in their expression in at least one of the included datasets under consideration of only entries with a p < 0.05. Up-regulated genes are colored in varying shades of red to illustrate their specific expression change. Down-regulated genes are colored in shades of green, with increasing intensity toward negative regulation. The Datasets were taken from: [McDonagh et al., 2008 (1); Schrettl et al., 2010 (2); Willger et al., 2008 (3)]. Pathway abbreviations: FA, fatty acid metabolism; AA, amino acid metabolism; SUC, sugar modifications; GSH, glutathione metabolism; GLU, gluconeogenesis*.

#### Target ranking and identification

The following top enzyme targets were predicted from this approach, zooming in on lipid metabolism and amino acid metabolism and considering only significantly differentially expressed genes (*p* < 0.05) and only strongly regulated genes (logFC > 1) and ranking all genes and potential targets accordingly.

Fatty acid synthases are down-regulated *in vivo* and under hypoxic conditions but up-regulated under iron deficiency conditions. In contrast, phosphoglycerate mutase and aspartokinase, and homoserine kinase (amino acid metabolism) are down-regulated *in vivo*, while they are up-regulated under hypoxia adaptation. Furthermore, in vitamin metabolism, thiamin biosynthesis proteins stand out, showing a negative regulation under *in vivo* condition (similar to the biofilm condition investigated in results, part 1). Finally, several cell wall carbohydrate metabolism genes stand out as strongly regulated and induced both for invasion and under hypoxia, such as glutathione S-transferase and the vacuolar transporter chaperone (Vtc4).

In summary, in this pipeline we show only targets according to one simple but clear criterion: High expression under infection-associated conditions as detailed above. However, other criteria help to identify alternative targets. As detailed in methods, we are currently screening for new targets with constant and high expression under several conditions, representing so-called housekeeping genes. As this involves analysis of much more transcriptome data sets, a definite list of metabolic target enzymes will be generated during future efforts.

### Targets from protein-protein interaction-based targeting of metabolism

**Figure 5:**
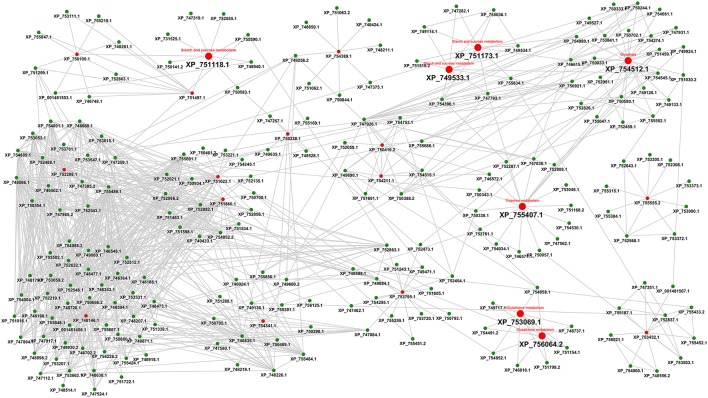
**Interactome view of metabolic proteins for antifungal targeting**. Red color nodes depict the network based drug targets in *A. fumigatus* interactome. The targets associated with the metabolism are shown with big red nodes with associated metabolic pathway annotation.

As a third line of research we considered an interactome-based approach. Figure 5 shows the protein-protein interaction-based drug targets.

The final interactome of *A. fumigatus* after considering the domain-domain interaction database (DDI) and localization filtering consists of 1903 nodes and 4743 PPIs and its interactome relation is given in Supplementary Table 9 (the interactome can also be analyzed in detail for hub proteins, bystanders, diameter, GO-categories etc., hence this data is made available here for such different research questions). The topologically important top 20% proteins were selected based on cumulative rank of hub degree rank and betweenness rank. Among these, 21 proteins were found to lack any significant similarity with human proteins, and are therefore proposed as preliminary network based drug targets (Table 3A). A subnetwork consisting of the proposed network based drug targets is shown in Figure 5. Out of 21 targets seven were annotated to be involved in metabolic pathways.

**Table 3:** **Results from two different ranking functions of the interactome pipeline^**a**^**.

**A**	**I**	**II**	**III**	**IV**			
**Gene**	**Degree Rank**	**Betweenness Rank**	**De+Be Rank**	**ReRanking**			**Annotatiom**
AFUA_6G11390	36	91	127	1			1,3-beta-glucanosyltransferase Gel2
AFUA_5G01960	8	119	127	1			Phosphate transporter (Pho88)
AFUA_1G09310	68	79	147	2			DUF6 domain protein
AFUA_3G11070	52	96	148	3			Pyruvate decarboxylase PdcA
AFUA_5G08420	124	55	179	4			High osmolarity signaling protein Sho1
AFUA_3G12800	137	60	197	5			Clathrin-coated vesiclec protein (Bud7)
AFUA_2G10740	94	108	202	6			Thiamin biosynthesis protein (Thi-4)
AFUA_5G12190	220	92	312	7			Transcription initiation factor subunit (TAF30)
AFUA_4G09660	19	310	329	8			Secretory component protein shr3
AFUA_2G12230	261	135	396	9			Mitochondrial large ribosomal subunit protein L16
AFUA_6G12400	261	142	403	10			1,3-beta-glucan synthase catalytic subunit FksP
AFUA_3G12320	307	117	424	11			Lipase/serine esterase
AFUA_1G06700	220	235	455	12			Metacaspase CasA
AFUA_3G14140	220	235	455	12			Metacaspase CasB
AFUA_4G13340	261	213	474	13			DUF907 domain protein
AFUA_2G17650	261	213	474	13			DUF907 domain protein
AFUA_2G17300	261	220	481	14			Glutathione S-transferase
AFUA_1G17010	261	220	481	14			Glutathione S-transferase
AFUA_2G09040	307	195	502	15			Vacuolar transporter chaperone (Vtc4)
AFUA_2G04010	220	302	522	16			Alpha, alpha-trehalose-phosphate synthase subunit
AFUA_6G12950	220	302	522	16			Trehalose-phosphate synthase/phosphatase complex subunit Tps1, putative
**B**		**I**	**II**	**III**			
**Gene**	**Priority order**	**New Rank (II**+**III)**	**RhumPDB RANK**	**Expression RANK**	**RhumPDB_score**	**Regulation, (Bertuzzi et al.**, **2014****)**	**Annotation**
AFUA_1G06940	1	11	1	10	183.000	UP	Chorismate synthase
AFUA_3G04210	2	40	1	39	183.000	–	Fatty acid synthase alpha subunit FasA
AFUA_2G10520	2	40	1	39	183.000	–	Urate oxydase UaZ
AFUA_2G06230	3	41	2	39	180.716	–	Glutamine amidotransferase:cyclase
AFUA_3G04220	4	43	4	39	178.380	–	Fatty acid synthase beta subunit
AFUA_3G09290	5	52	13	39	149.637	–	Phosphoglycerate mutase, 2,3-bisphosphoglycerate-independent
AFUA_6G04700	5	52	37	15	71.699	UP	Imidazoleglycerol-phosphate dehydratase
AFUA_3G06830	6	55	16	39	142.097	–	Aspartate-semialdehyde dehydrogenase
AFUA_1G14570	7	57	18	39	122.634	–	Phosphoribosyl-AMP cyclohydrolase
AFUA_5G06160	8	66	27	39	93.000	–	5-proFAR isomerase His6
AFUA_5G13130	9	77	38	39	70.079	–	Chorismate mutase
AFUA_2G10740	10	85	46	39	54.176	–	Thiamin biosynthesis protein (Thi-4)
AFUA_4G06460	10	85	83	2	5.301	UP	Pentafunctional AROM polypeptide
AFUA_3G14490	11	94	55	39	46.813	–	Ketol-acid reductoisomerase
AFUA_5G05590	12	102	48	54	52.301	DOWN	Aspartokinase
AFUA_1G05530	13	105	60	45	39.523	DOWN	Uridine nucleosidase Urh1
AFUA_5G05820	13	105	66	39	25.362	–	Homoserine kinase
AFUA_4G04030	14	109	70	39	14.660	–	Histidinol-phosphatase
AFUA_4G11980	15	114	63	51	33.665	DOWN	Anthranilate phosphoribosyltransferase
AFUA_6G12400	16	140	101	39	−0.513	–	1,3-beta-glucan synthase catalytic subunit FksP
AFUA_4G13680	17	167	117	50	−5000.000	DOWN	Phosphatidylserine synthase

*List of promising targets from two different ranking procedures. Table (A) describes the results of the first ranking calculation considering the genes degree rank (A.I) as well as its subsequent betweenness rank in the metabolic pathway (A.II). The added values from (A.I) and (A.II) are listed in (A.III). (A.IV) contains the re-ranking based on column (A.III). Table (B) proceeds with calculation of a RhumPDB Rank (B.II), an expression rank (B.III, Bertuzzi et al., 2014) and a final addition of those two values, leading to the new ranking (B.I). The resulting order illustrates the priority of the target in context with the metabolic function, the expression and the three dimensional structure of the protein. For detailed description see Materials and Methods*.

After consideration of the added Ranking scores from Degree Rank and Betweenness Rank and sorting of the resulting list we re-ranked the genes regarding their position. The five best hits include (1) 1,3-beta-glucosyltranferase Gel2 (AFUA_6G11390), (2) phosphate transporter (Pho88, AFUA_5G01960), (3) DUF6 domain protein (AFUA_1G09310), (4) pyruvate decarboxylase PdcA (AFUA_3G11070) as well as (5) high osmolarity signaling protein Sho1 (AFUA_5G08420).

In the subsequent analysis we determined a further ranking based on RhumPDB data and expression by calculation of the RhumPDB Score (Table 3B). The combination of those two values by adding them up lead us to a new rank. We then assigned them a priority order related to their position in the list. The five top hits resulting from this method are (1) chorismate synthase (AFUA_1G06940), (2) fatty acid synthase alpha subunit FasA (AFUA_3G04210), (3) urate oxydase UaZ (AFUA_2G10520), (4) glutamine amidotransferase:cyclase (AFUA_2G06230), and (5) fatty acid synthase beta subunit (AFUA_3G04220).

Hence, a total of 65 proteins were predicted to be important based on orthology with DEG(“database of essential genes”)-derived proteins.

Genes in the DEG database were included if they had scored to be essential for one organism under a specific condition. In general, essentiality depends very much on the environmental condition tested. Instead, the sequence similarity to the DEG entry can be determined by objective criteria. It is thus hard to predict essentiality of the *A. fumigatus* homolog. For the *A. fumigatus* genes we hence used DEG only to identify functionally conserved and probably important metabolic genes. Two preliminary targets identified in this way include the 50S ribosomal protein (AFUA_4G10480) and a cytochrome c oxidase family protein (AFUA_3G14440) that participates in oxidative phosphorylation.

#### Target ranking and identification

A total of 130 metabolism-associated *A. fumigatus* proteins were identified and subsequently parsed and prioritized regarding contained top targets (see Materials and Methods). The gene expression of predicted potential targets in the infection condition (Bertuzzi et al., 2014) was used to rank the genes according to their importance in infection and metabolism. The genes that were highly expressed at all four time point were given top priority (mean significant differential expression) then the gene highly expressed at three time points were ranked and so further on. A top expression rank represents a high gene expression at all time points during invasive infection (Supplementary Table 11). We also analyzed the RhumPDB score for targets and together with expression rank this score was used for prioritization. The score represents the normalized log10 ratio of BlastP *e*-value of *A. fumigatus* vs. human proteome and *A. fumigatus* vs. current PDB structures (Supplementary Table 12). The RhumPDB score orders the targets based on their higher closeness to crystallized PDB structures and their lack of similarity to human proteins. As a final reduction of the targets we implemented sequence similarity searches of *A. fumigatus* against human proteins by blast to avoid any off-targets. The genes/enzymes catalyzing the reactions that can also be catalyzed by alternative enzymes are indicated. Therefore, we also ignored such enzymes from our target list. The RhumPDB score and expression rank of the analyzed 130 metabolic proteins is listed in Supplementary Table 13. The final list consists of 22 proposed targets from fungal metabolic pathways (Supplementary Table 14).

As an example for a target list entry, PdcA (Pyruvate decarboxylase, AFUA_3G11070), participates in biofilm formation, which is represented by data from Muszkieta et al. (2013). PdcA is down-regulated three fold during biofilm formation. Hence, in principle, this target could also be picked up by a transcriptomics approach, but only if a different filter from our example is used, for instance strongly regulated genes, comparing wild type and biofilm formation. Other genes in our current list are not differentially expressed according to the Muszkieta dataset, so they are complementary found to those targets from the transcriptomic approach if using the filters just given.

The RhumPDB rank of this gene is 55, however, the protein is downregulated during invasive aspergillosis conditions and upregulated under iron limiting conditions, henceforth it might be possibly represent a valid target. AFUA_3G04210 (FasA Fatty acid synthase A) and AFUA_3G04220 (FasB Fatty acid synthase beta subunit) with RhumPDB rank of 1 and 4, respectively, significant expression changes, up-regulation in iron limiting condition and downregulation during invasion might be further promising targets. AFUA_3G14440 (cytochrome c oxidase family protein) from DEG can be examined as supported by expression changes under both tested conditions. These proteins would be the most interesting ones from this refined interaction-based pipeline to identify targets in the pathogen metabolism network (Figure 5).

In summary, we considered only top ranked proteins from each of the three sub-pipelines using interactomic data considering whether the target was connected to metabolism: either using structure and RhumPDB score (top seven targets all metabolic), or using combined degree rank and betweenness rank (top five targets given) as well as functional important, non-orthologous genes in the interactome (top two targets included).

### Identified top targets using expert knowledge and combined evaluation of the three pipelines

In general, however, detailed knowledge and extensive data support the target strategy, which is in fact essential for any new antifungal development. We show this now combining our three pipelines: metabolic pathway modeling and flux calculations, gene expression, and transcriptome data on metabolic adaptation as well as network modeling and protein interactions. All three focus on the following metabolic areas: (a) vitamin synthesis, (b) lipid synthesis, and (c) biosynthesis of amino acids. This focus was chosen as *A. fumigatus* differs in these pathways significantly from the host, which is of course a simplified search for metabolic targets. However, for a direct comparison of the three pipelines (and sub-pipelines) this focus is valid as it allows to combine and compare results from all three approaches pertaining to the same pathways (Table 4). The top seven genes where selected from the resulting list of the structure analysis methodology, calculating the RhumPDB score to assess the quality of the target suggestion. Using this calculation the most accurate results can be obtained (Table 4; Supplementary 14). All of the listed genes are involved in metabolic pathways.

**Table 4:** **Top Targets from protein-protein interaction-based targeting of metabolism^**a**^**.

	**Iron deficiency**	***In vivo***	**Hypoxia adaptation**	**Pathway**	**Annotation**
**Sup14**					
AFUA_3G04210	1.052	−1.058	−1.474	FA	Fatty acid synthase alpha subunit FasA
AFUA_3G04220	1.153	−1.971	n.s.	FA	Fatty acid synthase beta subunit
AFUA_3G09290	−0.706	−0.719	1.479	AA	Phosphoglycerate mutase, 2,3-bisphosphoglycerate-independent
AFUA_1G14570	n.s.	1.042	−2.718	AA	Phosphoribosyl-AMP cyclohydrolase
AFUA_4G06460	n.s.	2.834	n.s.	AA	Pentafunctional AROM polypeptide
AFUA_5G05590	n.s.	−1.060	1.703	AA	Aspartokinase
AFUA_5G05820	n.s.	−2.065	1.107	AA	Homoserine kinase
**Sup10**					
AFUA_4G09660	n.s.	1.849	n.s.		Secretory component protein shr3
AFUA_2G17650	−0.523	2.921	−2.795		DUF907 domain protein
AFUA_2G17300	n.s.	4.050	1.057	GSH	Glutathione S-transferase
AFUA_2G09040	n.s.	1.360	1.053	SUC	Vacuolar transporter chaperone (Vtc4)
AFUA_2G04010	n.s.	−1.125	1.182		Alpha, alpha-trehalose-phosphate synthase subunit
**ESSENTIAL GENES**					
AFUA_3G14440	0.916	−1.174	n.s.		Cytochrome c oxidase family protein
AFUA_4G10480	n.s.	−2.375	1.812		Mitochondrial large ribosomal subunit protein L30
logFC: -1	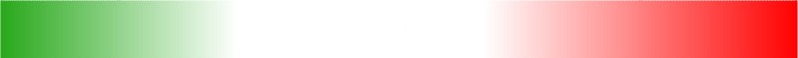			1	

a *Results of comparison of orthology results and transcriptomic results in consideration of metabolic function. Only differentially expressed genes with a confidence p < 0.05 are shown. Up-regulated genes are colored in varying shades of red to illustrate their specific expression change. The Datasets were taken from: [McDonagh et al., 2008 (1); Schrettl et al., 2010 (2); Willger et al., 2008 (3)]. Pathway abbreviations: FA, fatty acid metabolism; AA, amino acid metabolism; SUC, sugar modifications; GSH, glutathione metabolism; GLU, gluconeogenesis*.

Furthermore, we selected the top five of the resulting genes from network analysis strategy. Two of them are also known to participate in metabolic pathways. This methods accuracy is highly depending on the network structure to calculate valid targets but nevertheless could give two new metabolic targets (Table 4; Supplementary 10).

The remaining method of the pipeline is relevant for the assessment of essential genes with no human orthologs. The two best hits can be easily assigned to a metabolic pathway (Table 4; essential genes) and hence can be assumed as valid target candidates for antifungal therapy.

Using the results of the analysis for differentially expressed genes in three different datasets (McDonagh et al., 2008; Schrettl et al., 2010, accession GSE22052; Willger et al., 2008, accession GSE12376), we used a stringent cutoff of a *p*-value of 0.05 and a logarithmic fold change of >1.0 or respective < −1.0 (Table 4).

In a first approach we mapped the resulting genes from Supplementary Table 10 and Supplementary Table 14 onto the gene expression results from Supplementary Tables 6–8. In addition to the resulting list from Supplementary Tables 10, 14 we also included some genes which according to common knowledge (DEG, Luo et al., 2013) are considered as essential. Table 5 contains thus all genes that show differential expression values as well as significant regulation (logFC > 1; logFC < −1).

**Table 5:** **Singled out best targets from the combined pipeline regarding ***A. fumigatus***^**a**^**.

**Gene**	**Iron deficieny**	***In vivo***	**Hypoxia adapt**.	**Metabolic category**	**Evidence in literature**	**Genetic test worthwhile**	**Currently tested**	**Annotation**
**Sup6**								
AFUA_1G06940	0.000	0.820	−1.855	AA			19	Chorismate synthase (ARO2)
AFUA_3G04210	1.052	−1.058	−1.474	FA	1			Fatty acid synthase alpha subunit FasA
AFUA_2G10520	0.000	−0.757	−3.258	CAF	2.3			Urate oxydase UaZ
AFUA_2G06230	0.000	−1.210	0.000	AA	4			Glutamine amidotransferase:cyclase (HisH/F)
AFUA_3G04220	1.153	−1.971	0.000	FA	1			Fatty acid synthase beta subunit (FasB)
AFUA_3G09290	−0.706	−0.719	1.479	AA				Phosphoglycerate mutase, 2,3-bisphosphoglycerate-independent
AFUA_6G04700	0.000	−1.013	1.162	AA;GLU				Imidazoleglycerol-phosphate dehydratase (HisB)
AFUA_3G06830	0.000	−1.684	0.945	AA;GLU				Aspartate-semialdehyde dehydrogenase (HOM2)
AFUA_1G14570	0.000	1.042	−2.718	AA_HIS				Phosphoribosyl-AMP cyclohydrolase (HIS4)
AFUA_5G06160	0.000	NA	0.000	AA_HIS				5-proFAR isomerase (HIS6)
AFUA_5G13130	0.000	0.037	0.000	AA_a	5		19	Chorismate mutase (AroC)
^*^AFUA_2G10740	0.000	−1.164	0.000	THI				Thiamin biosynthesis protein (Thi-4)
AFUA_4G06460	0.000	2.834	0.000	AA_a				Pentafunctional AROM polypeptide (ARO1)
AFUA_3G14490	0.000	−1.401	−1.574	AA				Ketol-acid reductoisomerase (ILV5)
AFUA_5G05590	0.000	−1.060	1.703	AA_s				Aspartokinase (HOM3)
AFUA_1G05530	0.000	0.147	0.000	PYR				Uridine nucleosidase Urh1
AFUA_5G05820	0.000	−2.065	1.107	AA_s				Homoserine kinase (THR1)
AFUA_4G04030	0.000	NA	0.000	AA_HIS				Histidinol-phosphatase (HIS2)
AFUA_4G11980	0.000	0.083	0.000	AA_a				Anthranilate phosphoribosyltransferase (TRP4)
^*^AFUA_6G12400	0.000	−0.031	−1.568	GLU	6			1,3-beta-glucan synthase catalytic subunit FksP
AFUA_4G13680	0.000	−0.112	0.000	AA_s				Phosphatidylserine synthase
**Sup2**								
AFUA_6G11390	−0.926	0.459	−2.196		7			1,3-beta-glucanosyltransferase Gel2
AFUA_5G01960	−0.685	−0.353	−0.981		8			Phosphate transporter (Pho88)
AFUA_1G09310	0.000	0.021	0.000					DUF6 domain protein
AFUA_3G11070	0.987	−4.248	0.000	GLU				Pyruvate decarboxylase PdcA
AFUA_5G08420	−0.830	−0.213	0.000	MAPK	9			High osmolarity signaling protein Sho1
AFUA_3G12800	0.000	−0.645	0.000					Clathrin-coated vesiclec protein (Bud7)
^*^AFUA_2G10740	0.000	−1.164	0.000	THI				Thiamin biosynthesis protein (Thi-4)
AFUA_5G12190	0.000	−4.310	−0.817					Transcription initiation factor subunit (TAF30)
AFUA_4G09660	0.000	1.849	0.000					Secretory component protein shr3
AFUA_2G12230	0.000	−0.415	0.924					Mitochondrial large ribosomal subunit protein L16
^*^AFUA_6G12400	0.000	−0.031	−1.568	SUC	6			1,3-beta-glucan synthase catalytic subunit FksP
AFUA_3G12320	0.000	0.506	0.837					Lipase/serine esterase
AFUA_1G06700	0.000	−1.705	0.000		10			Metacaspase CasA
AFUA_3G14140	0.367	−1.273	−1.262		10			Metacaspase CasB
AFUA_4G13340	0.000	−0.008	−1.412					DUF907 domain protein (FlcA)
AFUA_2G17650	−0.523	2.921	−2.795					DUF907 domain protein (FlcA)
AFUA_2G17300	0.000	4.050	1.057	GSH				Glutathione S-transferase
AFUA_1G17010	0.000	0.681	0.000	GSH				Glutathione S-transferase
AFUA_2G09040	0.000	1.360	1.053					vacuolar transporter chaperone (Vtc4)
AFUA_2G04010	0.000	−1.125	1.182	SUC	11			Alpha, alpha-trehalose-phosphate synthase subunit (tpsA/B)
AFUA_6G12950	0.327	−0.773	0.000	SUC	11			Trehalose-phosphate synthase/phosphatase complex subunit Tps1
**ESSENTIAL GENES**								
AFUA_3G14440	0.916	−1.174	0.000					Cytochrome c oxidase family protein
AFUA_4G10480	0.000	−2.375	1.812					Mitochondrial large ribosomal subunit protein L30
**FURTHER CONSIDERED TARGETS**								
AFUA_2G15970	0.000	−1.561	0.000	PCS				Phosphatidylethanolamine N-methyltransferase
AFUA_5G05690	0.000	2.177	0.000	AA				Prephenate dehydratase
AFUA_1G02110	−0.832	0.086	0.000	AA_a				3-deoxy-7-phosphoheptulonate synthase
AFUA_7G04500	0.000	0.415	0.000	AA_HIS				ATP phosphoribosyltransferase
AFUA_3G11640	0.000	0.914	0.986	AA				Homoserine dehydrogenase
AFUA_4G10460	0.589	−0.266	2.419	AA				Homocitrate synthase
AFUA_5G07210	0.000	−0.588	1.909	AA				Homoserine O-acetyltransferase
AFUA_4G07360	−0.622	0.052	0.000	AA				Methionine synthase
AFUA_6G02860	0.000	1.827	1.566	GLU	12			2-methylisocitrate lyase
AFUA_6G03730	−0.668	2.433	1.286	AA				2-methylcitrate hydrolyase
AFUA_1G09050	0.000	−1.883	0.000	PCS	13			Methylene-fatty-acyl-phospholipid synthase
AFUA_4G12990	0.000	1.271	0.000		14–16			Thioredoxin reductase
AFUA_2G11290	−0.452	−0.573	0.000					Orotate phosphoribosyltransferase 1
AFUA_4G12600	0.000	−0.294	2.812					Phosphoribosylaminoimidazole carboxylase
AFUA_3G05650	0.000	0.474	1.158		17			Trehalose-phosphatase
AFUA_7G01220	0.000	−1.010	1.083					Squalene synthase
AFUA_5G10680	0.000	−0.203	0.000					Phosphomevalonate kinase
AFUA_3G05730	0.000	0.024	0.000					Nicotinate mononucleotide pyrophosphorylase
AFUA_5G08120	−0.402	3.931	1.030					Glutamate N-acetyltransferase
AFUA_2G10660	−1.133	−0.890	0.000		18			Mannitol-1-phosphate 5-dehydrogenas
logFC: -3	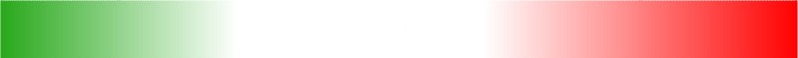			3				

* *Gene result in Sup10 + Sup14*.

a *The heat map visualizes the logarithmic fold change in expression of respective genes. Green color depicts a negative regulation compared to control, red color a positive. White cells in the heatmap are considered to show significant (p > 0.05) expression changes. Yellow colors indicate new promising targets. Mutants currently tested by us and thus already available are shown by a blue box. Genes already successfully tested contain numbered literature references: (1) Edwards et al., 1998, (2) Oestreicher et al., 1993, (3) Oestreicher and Scazzocchio, 1993, (4) Valerius et al., 2001, (5) Krappmann et al., 1999, (6) Dichtl et al., 2015, (7) Mouyna et al., 2005, (8) de Gouvêa et al., 2008, (9) Ma et al., 2008, (10) Richie et al., 2007, (11) Al-Bader et al., 2010, (12) Brock, 2005, (13) Tao et al., 2010, (14) Schrettl et al., 2010, (15) Bruns et al., 2010, (16) Shi et al., 2012, (17) Puttikamonkul et al., 2010, (18) Ruijter et al., 2003, (19) Sasse et al., 2016. Pathway abbreviations: FA, fatty acid metabolism; AA, amino acid metabolism (aromats: AA_a; serin: AA_s; histidine: AA_HIS); SUC, sugar modifications; GSH, glutathione metabolism; THI, thiamin biosynthesis; GLU, gluconeogenesis*.

Further targets which have no or only low orthology to human ones and additionally show high expression change in at least one condition (iron deficiency, *in vivo*/invasion initiation, hypoxia adaptation) are shown in the list. We also included two essential genes, AFUA_3G14440 and AFUA_4G10480. Additionally we considered enzymes which have metabolic pathway neighborhood to already known (according to literature and biochemical data) promising antimycotic targets, as targeting further members of such a known pathway promising to be targeted by an antimycotic may yield further attractive targets (Table 5).

#### Target ranking and identification considering all approaches

Table 5 only shows new targets remaining after selection in the pipeline and its major steps: (a) the metabolic pipeline, (b) the orthology analysis (Supplementary Tables 10, 14), and (c) the transcriptome dataset analysis (logFC >/< 1.0/-1.0; *p* < 0.05). The results of the metabolic model simulations are shown in an additional column, listing the pathway annotations of the proteins (as far as known) as well as their predicted activation (red: positive, green: negative). We show only the targets with a top rank which were still (after filtering as above) considered by our analysis to be worth testing experimentally. This list of 64 targets is thus not further ranked as in the interactome pipeline (ranking see above) but rather the top targets from all approaches are considered equally.

Nevertheless, this analysis result might be classified further. In particular, some of the new targets are already well described in literature (see references in Table 5) and published evidence (e.g., essential gene) supports them as promising targets. In those cases where such validating evidence exists, the entry was marked referring to the literature listed in the table legend. Further testing of those already known genes should next focus on identifying lead compounds to target them. The remaining genes were considered regarding their suitability as potential candidates by affiliation to any important metabolic pathway and supporting biological knowledge. The genes that preserved thus their potential are marked by a colored box in column (“genetic test worth doing”). Yellow labeled entries denote potential candidates, while blue labeled entries are candidates that currently are under our experimental evaluation (e.g., AFUA_3G09290). Furthermore, blue boxes containing a black dot indicates candidates studied with a conditional expression system in the course of our experimental evaluation (AFUA_1G06940, AFUA_2G10520, AFUA_6G04700, AFUA_5G13130). Moreover, two of those genes could already be verified regarding their importance for fungal *in vivo* growth and can be considered as promising potential drug targets (Sasse et al., 2016).

We hence see in Table 5 several potential targets, many being validated in literature: specifically some proteins connected to amino acid biosynthesis and fatty acid biosynthesis are already confirmed as good targets and well-studied (UaZ, AFUA_2G06230; AroC, AFUA_5G13130; FasA, AFUA_3G04210; FasB, AFUA_3G04220). Recent results validated our predictions regarding targets of the shikimate pathway (Sasse et al., 2016). The predicted and experimentally validated targets chorismate synthase (ARO2, AFUA_1G06940) and chorismate mutase (AroC, AFUA_5G13130) show a high potential as antimycotic. From our results we can even derive a more general strategy (see our Summary Table 5): Targeting specifically expanded protein families of the fungal pathogen that are connected to its metabolism. For *A. fumigatus* this is for instance a phosphate transporter like Pho88 (AFUA_5G01960), a protein participating in inorganic phosphate acquisition. In fact, Pho88 was already studied in *A. nidulans* as well as in *A. fumigatus* (de Gouvêa et al., 2008). Similarly, other branches of primary metabolism contain promising targets (Table 5), for instance another metabolic target would be to disrupt phospholipid biosynthesis by targeting genes like *cho1* (AFUA_4G13680) (Wolf et al., 2015).

AFUA_3G12320 lipase/serine esterase is another promising potential drug target. It has been studied in yeast only and it has no human homologs. Yeast homolog Lpl1 is nonessential and exerts phospholipase B activity to play a vital role in LD (lipid droplet) morphology, so its absence results in altered LD size (Selvaraju et al., 2014). However, as there is no auxotrophy and there might be redundancy with additional phospholipase B genes. However, from a drug point of view, a phospholipase B blocker would be considered interesting, as a drug, because it would probably block all phospholipase B activities in general, resulting in an inviable phenotype.

In contrast, the vacuolar transporter chaperone (Vtc4, AFUA_2G09040) is a vacuolar membrane polyphosphate polymerase; from *Candida* we know that this is a putative polyphosphate synthetase with decreased expression in hyphae compared to yeast-form cells; moreover, it is a fungal-specific protein (no human or murine homolog) with a typical virulence-factor like expression during infection. Most components of the pathway, except this protein, are shared in all organisms; yet, this gene itself is an essential gene target and might be explored further.

Furthermore, some members of the phospholipid biosynthesis like cho1 (AFUA_4G13680), cho2 (AFUA_2G15970), and choC (AFUA_1G09050) (Tao et al., 2010) are also worthwhile testing, in particular as ChoC has no human homolog. The deletion of choC in *A. nidulans* was studied by Tao et al. (2010): It results in highly restricted vegetative growth, swelling at the hyphal tips and the complete blockage of asexual and sexual development on culture medium lacking choline. If we extrapolate to the clinic, this may be a promising block against various *A. fumigatus* infection routes. However, in the lung, surfactant may help to counter balance such an antimycotic, as dipalmitoyl phosphatidylcholine (aka: lecithin) is a major component of pulmonary surfactant, and may provide an alternative source of phospholipids.

Finally, for this phospholipid pathway a promising, not yet explored strategy would be to target the connected enzyme flippase. Phosphatidylserine(s) are actively held facing the cytosolic (inner) side of the cell membrane by the enzyme. Specific blocking of flippase (including known *A. fumigatus* variants, e.g., Z5, DRS2 flippase) should inhibit *A. fumigatus* growth.

## Discussion

The strategy to target metabolism in fungal pathogens has been advocated previously (e.g., Tao et al., 2010; Sasse et al., 2016) while the combination of different omics strategies for antimycotic pipelines can provide efficiently novel targets in these pathways. The three approaches we combined are each whole fields of their own: metabolic modeling, transcriptome analysis and the study of PPI are here combined in a specific and novel way to systematically identify targets of the human-pathogenic mold *A. fumigatus*. Related work includes a detailed metabolic analysis (Li et al., 2013) to improve itaconic acid production in different Aspergillus species, and a first study of essential genes according to flux models in *A. fumigatus* (Thykaer et al., 2009). Regarding the latter, we present here our own detailed model on primary metabolism and are much stricter in validating potential targets by combining several approaches as well as testing and including direct genetic evidence and detailed transcriptome data. There are several exciting transcriptome studies in *A. fumigatus*, in particular studies identifying invasion-related gene expression changes (McDonagh et al., 2008; Willger et al., 2008; Schrettl et al., 2010) and detailed analyses of PPI in fungal infections (Lamoth et al., 2015).

The key to success in the identification of new antimycotics is efficient implementation. For the latter this paper provides a broad overview on available approaches, as three different pipelines are combined and evaluated against each other. We are not dogmatic about any of these approaches, for instance, starting from our calculations given (see Supplementary Material) the metabolic approach can be pushed much further to identify new pathways (Li et al., 2013), choke modes (Rahman and Schomburg, 2006), hub enzymes (Thadakamalla et al., 2005) and so on as visible in related work. The same applies for the transcriptome analysis (analyzing more and more different data-sets). A nice example is the analysis of gliotoxin production and attenuation in *A. fumigatus* (O'Keeffe et al., 2014). Analysis of PPI networks in *A. fumigatus* is particularly topical (Lamoth et al., 2015; Remmele et al., 2015). Hence, our interactome pipeline can be further refined profiting from new interaction data-sets which constantly appear online and in the literature. We hence suggest here that each of these pipelines has high potential in antimycotic target search, however, they are virtually complementary strategies to identify key metabolic targets and that is the underlying rationale why we combined them.

Nevertheless, there are some inherent limitations. To show that the different pipelines work and deliver, we were rather conservative and strict in our overall criteria: anything being homologous to humans (even low homology) was discarded as a potential drug target, only central metabolism was targeted and so on. Such an approach is good for delivering certified, strong targets and can be used to validate the different pipelines by knowledge (see Section Drug Targets with Orthology to Functionally Important Genes of the Results part). However, as the results confirm, this yields also several targets that have already been identified or are currently tested.

Hence, this has to be extended in future by more subtle approaches. A first measure will be to loosen the strict requirement of absent orthology to human proteins, allowing distant homologs. Furthermore, primary metabolism is defined by its connection and requirement for cellular growth (reason to pick it for antimycotic design), however, it needs not to be central in the metabolic web. In particular further areas connected to cell wall metabolism, virulence or, for instance, iron utilization can and will be scrutinized further. Finally, the metabolic calculations in particular, but also interaction screens and transcriptomics allow more refined screening methods: this includes synthetic lethality (hitting two targets that together stop fungal growth, calculation for instance by metabolic flux analysis), and exerting the full power of the transcriptomic approach by looking at ten or more conditions to identify all metabolic genes that are at least expressed under some specific condition. Furthermore, there can be better targeting of auxotrophic mutants, for instance by knowledge-based approaches and creating toxic metabolic intermediates which stop fungal growth in the fungus without harming the human patient. We believe that these more sophisticated approaches will allow an even better view on new antifungal strategies against *A. fumigatus* infection.

## Conclusions

Metabolism is interesting to target by novel antimycotic substances. Three different pipelines are made available here to investigate *A. fumigatus* in this respect. They validate potential targets from metabolism according to their importance in flux control and metabolic pathways, as key regulated enzymes under different challenging conditions or as central metabolic hubs with known protein structure, different from man, and, if possible, with some drug to target it. Together, these produced important targets, as validated by previous publications and currently ongoing experimental tests for about half of these. The others are made public here to allow further research and investigations. However, we followed a conservative approach demanding no homology to human proteins and avoiding more complex considerations such as targeting the same pathway twice or producing toxic intermediates. Future, more detailed analyses will follow up also these more sophisticated options. Additionally, the software and criteria applied can (and will) next be applied to other parts of the metabolic map in *A. fumigatus* and can also handle any other organism of interest if protein sequences are sufficiently comprehensively known.

## Author contributions

MK analyzed and calculated transcriptome and enzyme regulation data. MS and SG analyzed and calculated interactome-based data. CL calculated and analyzed metabolic fluxes and elementary modes. HH, NO, and SK and their PhD students AD, ZM, and JB, respectively, provided *A. fumigatus* expertise and experimental expertise on all protein targets suggested. TD analyzed data, lead and guided the study. MK and TD drafted and finalized the manuscript, all authors contributed expert advice, gave own comments, and agreed to the final submitted version of the manuscript.

## Funding

This study was supported by the German Federal Ministry of Education and Research (FKZ 031A408B to TD and 031A408A to SK), the Austrian Science Fund (FWF grant I1616 to HH), and the Israel Ministry of Health (MOH 3-0000-11080 to NO) by funding the AspMetNet consortium during the first call of the Infect-ERA research co-ordination action.

### Conflict of interest statement

The authors declare that the research was conducted in the absence of any commercial or financial relationships that could be construed as a potential conflict of interest.
