# Supplementary figures and images for: Systematic Identification of Anti-Fungal Drug Targets by a Metabolic Network Approach
Source: Front Mol Biosci. 2016 Jun 17;3:22. doi: 10.3389/fmolb.2016.00022 (PMC4911368; doi:10.3389/fmolb.2016.00022)

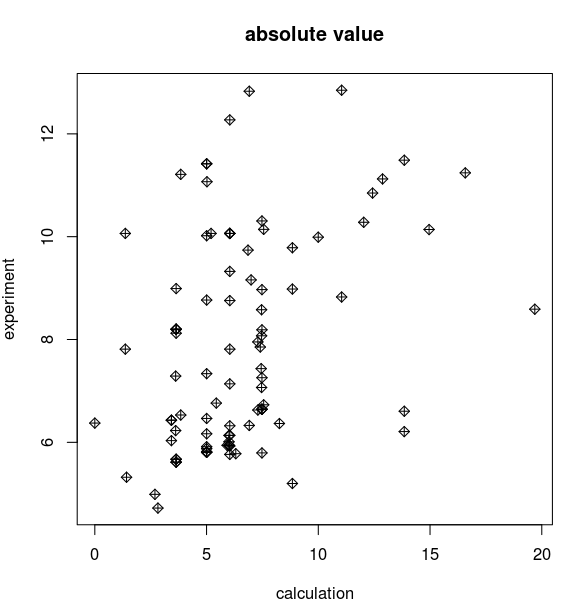

Supplement: Supplementary file 1 [file DataSheet1.ZIP › supplementary_figure_3_model_vs_exprs_wt.png]

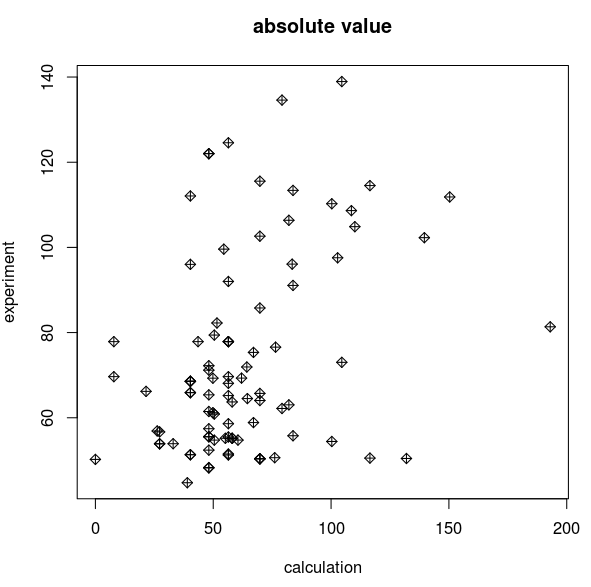

Supplement: Supplementary file 1 [file DataSheet1.ZIP › supplementary_figure_5_model_vs_exprs_biofilm.png]
